# Frequent detection of bocavirus DNA in German children with respiratory tract infections

**DOI:** 10.1186/1471-2334-6-109

**Published:** 2006-07-11

**Authors:** Benedikt Weissbrich, Florian Neske, Jörg Schubert, Franz Tollmann, Katharina Blath, Kerstin Blessing, Hans Wolfgang Kreth

**Affiliations:** 1 Institute of Virology and Immunobiology, University of Würzburg, Versbacher Str. 7, 97078 Würzburg, Germany; 2 Children's Hospital, University of Würzburg, Josef-Schneider-Str. 2, 97080 Würzburg, Germany

## Abstract

**Background:**

In a substantial proportion of respiratory tract diseases of suspected infectious origin, the etiology is unknown. Some of these cases may be caused by the recently described human bocavirus (hBoV). The aim of this study was to investigate the frequency and the potential clinical relevance of hBoV in pediatric patients.

**Methods:**

We tested 835 nasopharyngeal aspirates (NPA) obtained between 2002 and 2005 from pediatric in-patients with acute respiratory tract diseases at the University of Würzburg, Germany, for the presence of hBoV DNA. The specificity of positive PCR reactions was confirmed by sequencing.

**Results:**

HBoV DNA was found in 87 (10.3 %) of the NPAs. The median age of the infants and children with hBoV infection was 1.8 years (mean age 2.0 years; range 18 days – 8 years). Infections with hBoV were found year-round, though most occurred in the winter months. Coinfections were found in 34 (39.1 %) of the hBoV positive samples. RSV, influenza A, and adenoviruses were most frequently detected as coinfecting agents. Sequence determination of the PCR products in the NP-1 region revealed high identity (99 %) between the nucleotide sequences obtained in different years and in comparison to the Swedish viruses ST1 and ST2. An association of hBoV with a distinct respiratory tract manifestation was not apparent.

**Conclusion:**

HBoV is frequently found in NPAs of hospitalized infants and children with acute respiratory tract diseases. Proving the clinical relevance of hBoV is challenging, because application of some of Koch's revised postulates is not possible. Because of the high rate of coinfections with hBoV and other respiratory tract pathogens, an association between hBoV and respiratory tract diseases remains unproven.

## Background

Respiratory tract infections are a major cause of human morbidity and are caused by a broad spectrum of microbial agents. Viruses account for the largest number of respiratory tract infections. The so-called respiratory viruses include influenza virus A and B, parainfluenzae viruses, adenoviruses, respiratory syncytial virus (RSV), rhinoviruses, and coronaviruses. In recent years, several novel viruses have been discovered in patients with respiratory infections using molecular biology methods. These novel viruses include the human metapneumovirus and several coronaviruses (SARS, NL63, HKU1) [1,2]. The latest addition to this list was the human bocavirus (hBoV) described by Allander et al. [3]. Their screening method for unknown viral sequences in patient samples involved concentration of viral particles, nucleic acid isolation, random amplification of RNA and DNA, and finally sequencing and subsequent blasting of the amplified products. HBoV is most closely related to the minute virus of canines (MVC) and the Bovine Parvovirus (BPV), which have been classified in the genus *Bocavirus *within the *Parvoviridae *[4].

Classically, the postulates of Koch as modified by Rivers have been used to establish a causal relationship between viruses and a disease [5]. However, hBoV has not been propagated in cell culture, and there is no animal model so far. Therefore, proving the clinical relevance is challenging because application of some postulates of Koch and Rivers is not possible.

In the first description of hBoV, DNA was detected in 17 (3.1 %) of 540 Swedish children with lower respiratory tract disease. Three of the children were coinfected with other viruses (two with RSV and one with adenovirus). Because 14 hBoV-positive samples were negative for other respiratory viruses by standard screening, it was reasoned that hBoV may cause respiratory tract disease. In a second report of hBoV in Australian children and adults, hBoV was detected in 18 (5.6 %) of 324 respiratory samples. In ten of these (55.6 %), a coinfection with RSV (n = 8), hMPV or adenoviruses was detected [6]. In a third study from Japan, hBoV DNA was found in 18 (5.7 %) of 318 respiratory specimens of children with lower respiratory tract disease [7]. Samples positive for other viruses (RSV, influenza virus A and B, hMPV) were excluded from the study. Analysis of double infections was therefore not possible. In two more recent studies from Canada and France, hBoV DNA was detected in 18 of 1209 (1.5 %) and nine of 262 (3.4 %) respiratory samples, respectively [8,9]. In the Canadian study specimens of children and adults were tested. Specimens positive for other respiratory viruses were excluded. The French study examined samples of children below five years of age. Three of the nine hBoV DNA positive children (33.3 %) were coinfected with RSV.

In order to better understand the epidemiological pattern of hBoV infections and to analyze its clinical relevance, further studies on larger groups of patients are necessary. Therefore, we retrospectively tested nasopharyngeal aspirates of patients from the University of Würzburg Children's Hospital, Germany for the presence of hBoV DNA.

## Methods

### Samples

The samples tested for hBoV infection consisted of stored nasopharyngeal aspirates (NPA) that were sent by the University of Würzburg Children's hospital for screening of respiratory viruses from January 2002 to September 2005. On arrival in the viral diagnostic laboratory, the samples were routinely tested for the presence of respiratory virus antigens with an immunofluorescence assay (Respiratory Panel IFA Kit, Chemicon). The screening reagent of the kit detects antigens of adenoviruses, influenza viruses A and B, parainfluenza viruses 1 – 3, and RSV. NPAs showing positive reactions with the screening reagent were further studied by IFA using the seven single monoclonal antibodies contained in the screening reagent. Remaining NPA material was stored at -20°C until further testing for hBoV DNA. In addition to the samples from 2002 – 2005, a small number of samples (n = 17) from 1997 – 2001 were also available for retrospective testing. The study was carried out in compliance with the Helsinki declaration and was approved by the ethics committee of the medical faculty at the University of Würzburg.

### hBoV PCR and sequencing

DNA was extracted from 200 μl of the NPA samples using the High Pure Viral Nucleic Acid Kit (Roche, Mannheim, Germany) according to the instructions of the manufacturer. The elution volume was 50 μl. Amplification of hBoV DNA was performed with the NP-1 primers BoV188F (GAGCTCTGTAAGTACTATTAC) and BoV542R (CTCTGTGTTGACTGAATACAG) described by Allander [3] using the HotStarTaq DNA Polymerase (Qiagen, Hilden, Germany). PCR reactions were carried out in a 50 μl volume consisting of 5 μl extracted DNA, 1× Qiagen HotStar buffer, dNTPs at a final concentration of 200 μM each, 200 pmol of each primer, and 1.5 U of Taq Polymerase. The cycling conditions were 50 cycles (94°C 30 s, 53°C 40 s and 1 min at 72°C) after a preheating step of 10 min at 95°C.

After amplification, PCR products were visualized by staining with ethidium bromide on agarose gels. A PCR reaction was considered as positive when a band of the expected size (354 base pairs) was visible. To confirm the sequence specificity, all PCR products from positive reactions were sequenced completely in both directions using Big Dye terminator chemistry and the ABI Prism 3100 (Applied Biosystems, Darmstadt, Germany).

General laboratory procedures to prevent PCR contamination were strictly adhered to. One negative control was extracted and amplified for every five NPA samples. All negative controls were found to be negative for hBoV DNA. A plasmid containing the PCR product cloned in the vector pCR^®^2.1-TOPO^® ^(Invitrogen) was used as positive control. The sensitivity of the hBoV assay was approximately 10 copies per reaction.

## Results

**Figure 1:**
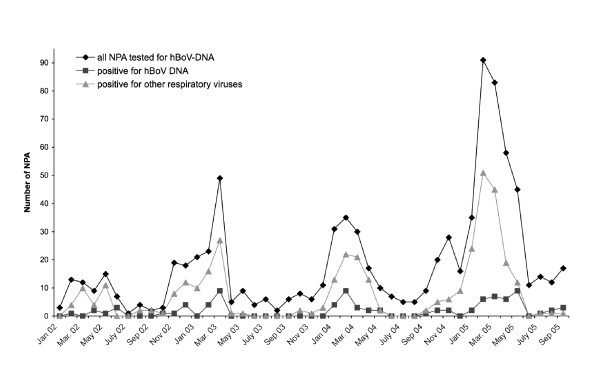
**Temporal distribution of hBoV infections**. Temporal distribution of the hBoV DNA positive NPAs compared with the total number of NPAs received and with the total number of positive results by immunofluorescence staining for antigen of respiratory viruses (RSV, influenza A/B, parainfluenza 1/2/3, adenoviruses).

From January 2002 to September 2005, 901 nasopharyngeal aspirates (NPA) of 786 hospitalized infants and children with febrile respiratory tract diseases were received for viral diagnostic evaluation. The median age of the patients was 1.6 years (mean age 3.4 years; range 12 days – 16 years), and 59 % were boys. The seasonal distribution of all samples is shown in Figure 1. Because of insufficient volume of the stored material, 66 samples had to be excluded from the retrospective testing of hBoV DNA. The median age and the seasonal distribution of the hospitalization were not significantly different between the patients with and without sufficient NPA sample volume.

By routine immunofluorescence testing for antigen of respiratory viruses, a positive diagnosis was obtained for 357 (39.5 %) of the NPA samples. RSV (n = 158; 17.4 %) and influenza A virus (n = 98; 10.9 %) were most frequently found. Further details are shown in Table 1. Coinfections with two or more viruses were detected in 7 of the cases using the antigen assay.

**Table 1:** Frequency of detection of respiratory viruses and of hBoV in NPA samples

	Total population n	Positive results n	Males
**Results of IFA testing for viral antigen**	901		59.0 %
Total positive results by IFA	901	357 (39.5 %)	55.5 %
• RSV	901	158 (17.4 %)	52.5 %
• Influenza A	901	98 (10.9 %)	57.1 %
• Adenovirus	901	53 (5.9 %)	62.3 %
• Parainfluenza 1/2/3	901	39 (4.3 %)	48.7 %
• Influenza B	901	16 (1.8 %)	50.0 %
• Coinfections	901	7 (0.8 %)	14.3 %
			
**Results of hBoV PCR**	835	87 (10.3 %)	59.8 %
hBoV positive with coinfections	87	34 (39.1 %)	58.8 %
• with RSV	87	14 (16.1 %)	
• with influenza A	87	9 (10.3 %)	
• with adenovirus	87	9 (10.3 %)	
• with parainfluenza 1/2/3	87	1 (1.1 %)	
• with influenza B	87	1 (1.1 %)	

**Figure 2:**
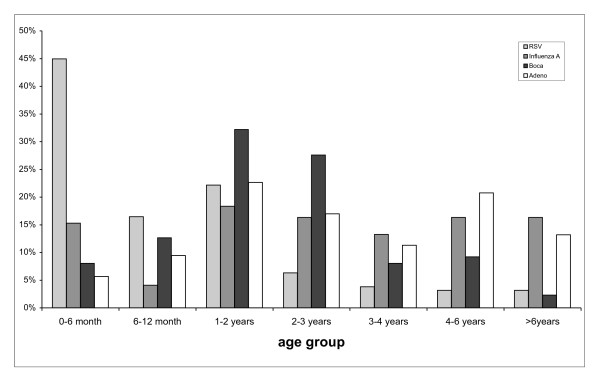
**Age distribution of hBoV infections**. Age distribution of hBoV infected children compared with the age distribution of children infected with RSV, influenza A, or adenoviruses.

Of the 835 NPAs tested for hBoV DNA, 87 (10.3%) samples were found to be positive by PCR and subsequent sequencing. The male to female ratio of the hBoV positive infants and children (59.8 % boys) was similar to the ratio in the population tested. Their median age was 1.8 years (mean 2.0 years; range 18 days – 8 years). The age distribution of the hBoV positive patients and of the RSV, adenovirus and influenza A virus positive children for comparison is shown in Figure 2. Median ages were significantly different for these four infectious agents, except for the comparison between influenza A and adenovirus infections (Table 2). While infections with RSV peaked during the first six months of life (median 0.7 years), most hBoV infections (n = 52; 59.8 %) occurred at the age of 1 – 3 years. Infections with influenza A virus were more evenly distributed over a wider age range (median 2.7 years).

**Table 2:** Median age of patients with viral respiratory tract infections

			p-values^1 ^for groupwise comparison		
Infection with	n	Median age	RSV	Adeno	Influenza A
hBoV	87	1.8	p < 0.0001	p = 0.0046	p = 0.0031
RSV	158	0.7		p < 0.0001	p < 0.0001
Adenovirus	53	2.7			p = 0.8
Influenzavirus A	98	2.4			

^1^by Mann-Whitney-test

**Table 3:** Seasonal frequency of hBoV infections

Period^1^	NPA samples tested n	hBoV positive NPAs n (%)
Jan 02 – June 02	59	7 (11.9%)
July 02 – June 03	159	19 (11.9%)
July 03 – June 04	169	19 (11.2%)
July 04 – June 05	405	36 (8.9%)
July 05 – Sep 05	43	6 (14.0%)

^1^Observation periods were chosen from summer to summer whenever possible in order to include the complete winter season

Infections with hBoV were found year-round, though most occurred in the winter months (Figure 1). The shape of the curves of the total number of NPA samples received and of the hBoV positive samples appeared to be almost in parallel. There were no significant differences between the yearly frequencies of hBoV positive results between 2002 and 2005 (Table 3). In 17 samples that were available from before 2002, HBoV was retrospectively found in 3 samples, two from 1998 and one from 2001.

In 34 (39.1 %) of the hBoV positive infants and children, coinfections with other respiratory viruses were present, most frequently with RSV (n = 14) followed by influenza A (n = 9). The percentage distribution of the coinfecting agents among the hBoV positive samples was similar to the distribution of these agents in the total population (Table 1).

Sequence determination of the hBoV PCR products (NP-1 region of the genome) revealed high identity (99 %) between the nucleotide sequences obtained in different years and in comparison to the Swedish viruses ST1 and ST2.

Clinical data were available for 63 of the 87 hBoV positive NPAs. The patients suffered from upper and/or lower respiratory tract diseases (Table 4). Associations between all hBoV infections and distinct clinical manifestations were not apparent. Because of the retrospective nature of the study and because clinical data for the hBoV negative samples were not obtained, a statistical analysis of this aspect was not possible. When the clinical diagnoses of the children with and without coinfections with other respiratory pathogens were compared, pneumonia was found more often in the group of children without coinfections (Table 4). This association was borderline significant (p = 0.044 by Fisher's exact test).

**Table 4:** Clinical manifestations in 63 hBoV DNA positive infants and children with and without coinfections^1^

Diagnosis at discharge from hospital	n with coinfection	n without coinfection	total n (%)
Upper respiratory tract disease (rhinitis, otitis media, tonsillitis, pharyngitis, laryngotracheitis, apnoic spells)	12	13	25 (39.7 %)
			
Lower respiratory tract disease (total)	8	24	32 (50.8 %)
• bronchitis	3	7	10 (15.9 %)
• wheezing bronchitis	4	5	9 (14.3 %)
• bronchiolitis	0	2	2 (3.2 %)
• pneumonia	1	10^2^	11 (17.5 %)
			
Febrile seizures	3	3	6 (9.5 %)

^1^Clinical information was available for 63 of the 87 hBoV DNA positive samples

^2^p = 0.044 by Fisher's exact test

## Discussion

We found hBoV DNA in 10.3 % of NPA samples obtained from infants and children with respiratory tract diseases during the years 2002 to 2005 in the region of northern Bavaria in Germany. This is the highest frequency reported so far. To our knowledge, there have been five previous reports on hBoV infections, the original report from Sweden with a frequency of 3.1 %, and reports from Australia, Japan, Canada and France with frequencies of 5.6 %, 5.7 %, 1.5 %, and 3.4 %, respectively. In our study, hBoV infections were almost as frequently found as infections with influenza A virus, the second most common respiratory infection, and they were considerably more frequent than infections with influenza B, parainfluenzae, and adenoviruses. However, in contrast to the PCR method used for hBoV detection, the other respiratory viruses in our study were examined by IFA. When comparing the detection frequencies, this difference in detection methods has to be taken into consideration. In general, PCR assays are more sensitive than antigen detection methods [10]. Therefore, it is likely that the true prevalence of the respiratory viruses that were analyzed by IFA is actually higher than here reported.

There are several possible explanations for the higher frequency of hBoV infections observed in our study compared to the previous reports. Firstly, the difference may be due to an increased sensitivity of our PCR assay. In all previous studies as well as in ours, single round hot-start PCRs have been employed to detect hBoV DNA. However, the assays vary in the number of PCR cycles performed (35 cycles [3,7-9]; 45 cycles [6]; 50 cycles (present study)). Depending on the assay optimization, 35 cycles may not be sufficient to detect weakly positive samples. In addition, data on the assay sensitivity were not provided in either of the previous studies. Using a plasmid control, we were able to show that our assay regularly detects approximately 10 copies of hBoV DNA per reaction. In order to obtain information on the amount of hBoV DNA present in the NPAs and in other secretions, a real-time PCR assay is currently under development.

A second potential reason for differing infection frequencies between studies may be due to regional and temporal differences in the incidence of hBoV infection. In contrast to the previous reports, which have studied samples from only one or two winter seasons, the NPAs in our studies have been collected during four consecutive years. In general, seasonal differences of sample acquisition may account for varying incidence numbers. In our study, for example, proportions of influenza A, parainfluenza 3, and adenovirus infections were considerably different between winter seasons (data not shown). However, this was not the case for hBoV infections. In four consecutive winter seasons, we observed a similar frequency of approximately 10 %. Therefore, seasonal variation is unlikely to account for the observed high frequency of hBoV infections in our study population. So far, hBoV has been detected in Sweden, Australia, Japan, Canada, France, and Germany, and it appears that hBoV has a worldwide distribution. It remains to be determined how incidence numbers are influenced by regional aspects.

Thirdly, the higher frequency observed in our study may be related to different patient populations. Children hospitalized for respiratory tract diseases were included in all studies published so far [3,6-9]. Two studies additionally examined adults and outpatients [6,8]. While three studies as well as ours included patients with upper and/or lower respiratory tract disease [6,8,9], two studies focused on patients with lower respiratory tract diseases [3,7]. Thus, it is difficult to compare patient populations in the hBoV studies.

In agreement with the previous reports [3,6-9], sequencing of the PCR products in the NP-1 region revealed a nucleotide identity of more than 99 % between different samples. This was also true for the two hBoV DNA positive samples from 1998. Although much more sequence information on hBoV will be required, the data available so far indicate that hBoV may be a highly conserved virus.

The age distribution of hBoV infections found in our study is similar to previous reports [3,6-9]. Most hBoV infections occurred between 6 months and 3 years of age. This distribution is compatible with protection from infection by maternal antibodies in the first year of life. Future studies of the seroprevalence of hBoV antibodies in different age groups will shed light on this issue.

Analysis of a potential association between hBoV infection and clinical manifestations is limited by the retrospective nature of our study, by the high number of double infections, and by the fact that clinical information was obtained only for hBoV positive patients. However, it seems that there is no obvious association between hBoV infection and a distinct clinical manifestation. Instead, a broad spectrum of both upper and lower respiratory tract diseases was observed. When clinical diagnoses of hBoV DNA positive patients with and without coinfections were compared, pneumonia was found more frequently in children without coinfection. However, this association of borderline significance (p = 0.044) should be regarded with caution because of small numbers. If pneumonia was caused by hBoV infection, it is unclear how a coinfection could result in a less frequent manifestation of this disease.

The assumption in the first description of hBoV, that this virus might be an etiologic agent of respiratory tract disease, was based on the fact that hBoV infections were found significantly more often in samples negative for other respiratory viruses. However, with coinfection rates ranging fom 33.3 % to 55.6 %, these findings were neither confirmed by the other studies that analyzed coinfections [6] nor by us. The true number of coinfections in our study is probably even higher than the reported 39.1 %, because antigen-based methods were used for screening of respiratory viruses other than hBoV, and because several respiratory pathogens such as coronaviruses, rhinoviruses, enteroviruses and the human metapneumovirus were not tested for. On the basis of the considerable number of coinfections, one might argue that hBoV is an aggravating factor of respiratory diseases, an innocent bystander that is just detected by chance, or a persisting virus that is reactivated by the inflammatory process.

Thus, it is uncertain at present, if hBoV is indeed an etiologic agent of respiratory tract (or other) diseases. Several viruses detected by molecular biology methods in recent years are still in search for a relevant clinical disease [11,12]. If hBoV has to be added to this list remains to be determined.

## Conclusion

HBoV is frequently found in NPAs of hospitalized children with acute respiratory tract diseases. Proving the clinical relevance of hBoV is challenging, because application of some postulates of Koch is not possible. Because of the high rate of coinfections with hBoV and other respiratory pathogens, an association between hBoV and respiratory tract diseases remains unproven.

## Competing interests

The author(s) declare that they have no competing interests.

## Authors' contributions

BW and HWK designed and coordinated the study. FN performed the hBoV DNA testing. HWK, KBlessing and KBlath collected clinical data. BW, JS and FT collected virological data. All authors participated in the data analysis. BW and FN drafted the manuscript. All authors read and approved the final version of the manuscript.

## Pre-publication history

The pre-publication history for this paper can be accessed here:


